# Role of MS4A7 in Regulating Microglial Polarization and Neuroinflammation in Spinal Cord Injury via the cGAS‐STING‐NLRP3 Axis

**DOI:** 10.1111/cns.70390

**Published:** 2025-06-16

**Authors:** Xiangrui Li, Junpeng Liu, Youliang Deng, Fang Xing, Xihua Lu, Zhen Zhang, Changsheng Li

**Affiliations:** ^1^ Department of Anesthesiology The Affiliated Cancer Hospital of Zhengzhou University & Henan Cancer Hospital Zhengzhou China; ^2^ Department of Anesthesiology Xinqiao Hospital, the Third Military Medical University Chongqing China; ^3^ Department of Anesthesiology The Third Affiliated Hospital of Zhengzhou University Zhengzhou Henan Province China

**Keywords:** cGAS‐STING pathway, inflammasome, microglial polarization, motor function, MS4A7, pain, spinal cord injury

## Abstract

**Background and Objectives:**

Spinal cord injury (SCI) leads to debilitating neurological deficits primarily due to the inflammatory response triggered by secondary injury mechanisms. Microglial activation and polarization significantly influence this response, with pro‐inflammatory (M1) polarization exacerbating damage and anti‐inflammatory (M2) polarization promoting repair. MS4A7, a membrane‐bound protein involved in immune regulation, has been implicated in inflammation, but its role in SCI remains unexplored. This study investigates the function of MS4A7 in modulating microglial polarization and its downstream effects on the inflammatory response in SCI, focusing on the cGAS‐STING‐NLRP3 axis.

**Methods:**

A combination of in vivo and in vitro approaches, including mouse SCI models and BV2 microglial cells, was employed. Differential gene expression analysis was conducted using the GSE93561 dataset. MS4A7 expression was modulated using shRNA and overexpression plasmids. Microglial polarization was assessed via immunofluorescence, RT‐qPCR, and ELISA for M1 (iNOS, IL‐1β, TNF‐α) and M2 (Arg1, IL‐10, CD206) markers. Pyroptosis and inflammasome activation were examined using PI staining, LDH release, and NLRP3/GSDMD assays. The role of the cGAS‐STING pathway was evaluated using activators (diABZI) and inhibitors (C‐176), and NLRP3 inflammasome activity was pharmacologically inhibited with MCC950.

**Results:**

MS4A7 was significantly upregulated in SCI tissues (*p* < 0.01). Knockdown of MS4A7 reduced M1 markers (iNOS, IL‐1β, and TNF‐α) and increased M2 markers (Arg1, IL‐10, and CD206), promoting anti‐inflammatory polarization (*p* < 0.05). Conversely, MS4A7 overexpression enhanced M1 polarization and pyroptosis through the NLRP3 inflammasome. In vivo, MS4A7 knockdown improved locomotor recovery (BMS score, *p* < 0.05) and alleviated pain‐related behaviors (PWL and PWT, *p* < 0.01). The cGAS‐STING pathway mediated NLRP3 activation, with pharmacological inhibition mitigating pro‐inflammatory effects and favoring tissue repair.

**Conclusions:**

In this study, we found that MS4A7 exacerbates inflammation and promotes M1 polarization via the cGAS‐STING‐NLRP3 axis in SCI. Targeting MS4A7 and its associated pathways offers potential therapeutic strategies to mitigate neuroinflammation and enhance recovery. These findings provide new insights into the molecular mechanisms underlying SCI pathophysiology and highlight MS4A7 as a promising therapeutic target.

## Graphical abstract

MS4A7 promotes M1 microglial polarization and neuroinflammation in SCI via the cGAS‐STING‐NLRP3 axis, highlighting it as a therapeutic target for neuroprotection and recovery enhancement. Downregulation of MS4A7 helped alleviate pain‐related behaviors and improve locomotor recovery.

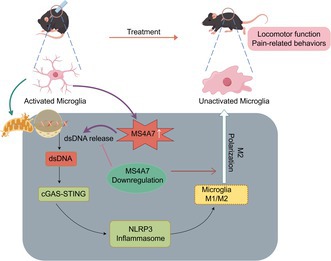

## 1 Introduction

Spinal cord injury (SCI) is a devastating condition with profound consequences on the physical, psychological, and social well‐being of affected individuals. The severity of SCI often leads to permanent neurological impairment, with the extent of disability determined by the injury's location and degree of damage. A major factor influencing SCI prognosis is the inflammatory response, which exacerbates the initial injury and contributes to tissue damage [1]. SCI involves both primary and secondary injury mechanisms, with the primary injury resulting from mechanical trauma and the secondary injury resulting from a cascade of biochemical and cellular events [2]. This secondary damage triggers a series of amplified inflammatory responses, leading to further neuronal death, glial activation, and demyelination, all of which contribute to worsening neurological deficits [3]. Despite substantial advances in SCI research, the precise molecular and cellular mechanisms driving its occurrence and progression remain poorly understood. Understanding these mechanisms is crucial for developing effective therapeutic strategies to promote recovery and improve the quality of life for SCI patients, and therefore warrants further investigation.

Microglia are the resident macrophages of the central nervous system (CNS) and play a crucial role in maintaining homeostasis and responding to injury [4]. Under normal conditions, microglia are involved in the surveillance and regulation of the neuronal environment, but upon SCI, they become rapidly activated [5]. Activation of microglia triggers a complex response that includes the release of pro‐inflammatory cytokines, chemokines, and reactive oxygen species, all of which contribute to secondary damage following SCI [5]. Microglial activation is accompanied by a process known as microglial polarization, where they adopt distinct phenotypes based on the microenvironment and signaling cues [6]. Microglia can polarize into two primary phenotypes: the pro‐inflammatory M1 phenotype, which exacerbates tissue damage, and the anti‐inflammatory M2 phenotype, which promotes tissue repair and neuroprotection [6]. The balance between these polarized states is crucial for modulating the inflammatory response post‐SCI. While excessive M1 polarization can intensify neuronal damage and impair recovery, M2 polarization has been shown to attenuate inflammation and foster tissue regeneration [7]. Understanding the mechanisms that govern microglial polarization and their subsequent impact on SCI‐induced inflammation is vital for identifying therapeutic targets that can mitigate secondary injury and enhance recovery after SCI.

The MS4A7 gene belongs to the MS4A (membrane spanning 4‐domains, subfamily A) family, which encodes a group of proteins characterized by their four transmembrane domains and specific structural features [8, 9]. MS4A7 is a membrane‐bound protein that is primarily expressed in immune cells such as microglia and macrophages [10]. It is involved in various cellular processes, including signal transduction, immune responses, and inflammation regulation [9]. Recent study has highlighted the role of MS4A7 in modulating the inflammatory response, especially in microglia and macrophages [10]. In these cells, MS4A7 appears to regulate the balance between pro‐inflammatory and anti‐inflammatory responses, which is critical in diseases involving inflammation [10]. Beyond its involvement in inflammation, MS4A7 has also been implicated in several diseases, including neurodegenerative disorders, cancers, and metabolic conditions. Recent research suggests that MS4A7 is highly expressed in tumor‐associated macrophages (TAMs), where it modulates the tumor microenvironment and immune evasion, potentially influencing cancer progression [9, 11]. In the central nervous system, MS4A7 has been linked to microglial function, indicating a potential role in neuroimmune interactions [12]. Additionally, MS4A7 has been reported to regulate diet‐induced metabolic dysfunction‐associated steatohepatitis through the modulation of NLRP3 inflammasome activation [13]. Although MS4A7 has been implicated in several inflammatory‐related diseases, such as autoimmune conditions and neurodegenerative disorders, its specific role in SCI remains unexplored. Despite the growing body of evidence supporting its involvement in other diseases, the potential impact of MS4A7 in SCI pathophysiology and its mechanisms of action in SCI‐induced inflammation are yet to be determined.

The cyclic GMP‐AMP synthase (cGAS)‐stimulator of interferon genes (STING) pathway is a critical component of the innate immune response, responsible for detecting cytosolic DNA and triggering type I interferon production [14]. Upon DNA binding, cGAS catalyzes the synthesis of cyclic GMP‐AMP (cGAMP), which binds to STING, activating downstream signaling cascades that lead to the production of inflammatory cytokines [15]. This pathway plays a key role in the regulation of inflammation and has been implicated in various immune‐related diseases. Recent studies have highlighted the importance of the cGAS‐STING pathway in the modulation of inflammation [15, 16]. It is now recognized as a central regulator of the inflammatory response in several conditions, including autoimmune diseases and cancer. Recent studies have identified the cGAS‐STING pathway as a crucial sensor of cytosolic DNA, primarily linking innate immune activation to neuroinflammatory diseases [17]. This pathway has been extensively studied in conditions such as Alzheimer's disease, Parkinson's disease, and multiple sclerosis, where its activation contributes to chronic neuroinflammation and neuronal damage [18, 19, 20]. However, emerging evidence suggests that the cGAS‐STING axis may play a pivotal role in SCI due to its ability to detect cellular damage‐associated molecular patterns (DAMPs), such as mitochondrial DNA (mtDNA) released following traumatic spinal cord injury [21]. In macrophages and microglia, the cGAS‐STING pathway has been shown to enhance cytokine secretion, contributing to the inflammatory environment [22]. Activation of this pathway also influences the polarization of macrophages toward pro‐inflammatory phenotypes, further amplifying the immune response [23]. Given the well‐established role of neuroinflammation in secondary injury after SCI, the involvement of cGAS‐STING in this process is highly plausible. While inflammation is a hallmark of SCI and contributes to secondary injury, no direct evidence has yet elucidated the role of the cGAS‐STING pathway in modulating the MS4A7‐associated inflammatory response following SCI. Further investigation is needed to understand whether this pathway plays a crucial role in SCI‐related inflammation and its potential mechanisms.

The primary aim of this study is to investigate the role of MS4A7 in regulating the inflammatory response following SCI and to elucidate the underlying mechanisms. Given the critical involvement of inflammation in secondary damage after SCI, understanding the function of MS4A7 in this context could provide valuable insights into the modulation of inflammatory pathways. By exploring how MS4A7 influences microglial activation, polarization, and the subsequent inflammatory cascade, this research may identify novel therapeutic targets for modulating inflammation and improving recovery outcomes in SCI.

## 2 Methods and Materials

### 2.1 SCI Model

In this study, female C57BL/6J mice, aged 8 weeks, were used as the experimental model for SCI. All mice were housed in a temperature‐controlled facility (22°C ± 2°C) under a strict 12‐h light–dark cycle, with ad libitum access to food and water. The housing environment was carefully regulated to minimize external stressors that could influence post‐injury recovery. Mice were also housed in standard cages with appropriate enrichment to reduce variability in locomotor function assessments. All animal experiments were conducted in accordance with institutional animal care guidelines and approved by the Ethic Committee of Zhengzhou University. For the SCI procedure, a contusion injury was induced at the thoracic (T10) level of the spinal cord using the impactor system from a height of 6.5 cm with a successful rate of 95% (5 g, RWD, Shenzhen, Guangdong, China). Post‐injury, animals were monitored weekly for signs of recovery, including motor function and allodynia. All experiments were designed and reported according to the Animal Research: Reporting of In Vivo Experiments (ARRIVE) guidelines.

### 2.2 BV2 Cell Culture and Treatment

BV2 cells, a widely used mouse‐derived microglial cell line, were selected for this study due to their well‐characterized polarization responses and ease of manipulation in vitro [24, 25]. BV2 microglial cells were cultured in DMEM (Dulbecco's Modified Eagle Medium, Gibco) supplemented with 10% fetal bovine serum (FBS, Gibco) and 1% penicillin–streptomycin (Servicebio) in a humidified incubator at 37°C with 5% CO_2_. For all experiments, cells were passaged at 80%–90% confluence. To ensure consistency across experiments, cells were seeded at a uniform density of 2 × 10^6^ cells per well in 6‐well plates or 1 × 10^5^ cells per well in 24‐well plates and allowed to adhere for 24 h. Following adherence, cells were treated with lipopolysaccharide (LPS, 1 μg/mL) and adenosine 5′‐triphosphate (ATP, 5 mM) for 24 h to induce an inflammatory response or vehicle (PBS) as control. All experiments were performed in triplicate and repeated independently.

### 2.3 Basso Mouse Scale

Hindlimb motor function was assessed using the Basso mouse scale (BMS) at baseline (prior to surgery) and on postoperative Days 1, 3, 7, 14, 21, and 28, as described by Basso et al. [26]. The BMS scoring system evaluates locomotor recovery in mice, assigning scores ranging from 0 to 9, where 0 represents complete absence of ankle movement and 9 denotes full functional recovery. Scoring was performed by two independent, blinded observers to minimize bias and ensure consistency in the evaluation process. The final score for each mouse was determined by averaging the scores given by both assessors.

### 2.4 Louisville Swim Scale

Hindlimb function, trunk stability, and body angle were assessed using the Louisville swim scale (LSS) prior to injury and at 7, 14, 21, and 28 days post‐SCI, as described by Smith et al. [27]. The LSS assigns scores ranging from 0 to 15, with 0 indicating no hindlimb movement and 15 representing normal hindlimb movement. To ensure acclimatization to the swimming environment, all mice underwent pre‐training sessions before the assessment. Scoring was conducted by two independent, blinded observers to enhance reliability, and the average score for each mouse was calculated and used for statistical analysis.

### 2.5 Footprint Analysis

Footprint analysis was conducted at 7, 14, 21, and 28 days post‐SCI following the protocol described by de Medinaceli et al. [28]. Nontoxic paint was applied to the plantar surfaces of the limbs, with red for the forelimbs and green for the hindlimbs. Mice were then placed in a confined walkway lined with paper to record their footprints. Trials were repeated if a mouse turned around before completing the walkway. Quantitative parameters, including stride length (distance from the start to the endpoint of the hind paw), angle of rotation (angle between the third toe and the stride line), and interlimb coordination (distance between ipsilateral forelimb and hindlimb placement), were analyzed. Each mouse underwent three trials per time point, and the average value for each parameter was used for analysis.

### 2.6 Formalin Pain Test

The formalin pain test was performed to evaluate pain behavior in mice [29]. Mice were first acclimatized to the testing environment by placing them individually in a transparent cylindrical jar (9.5 cm in diameter and 10 cm in height) for 5 min. Following acclimation, 20 μL of 2% formalin solution was injected subcutaneously into the plantar surface of the right hind paw. Pain responses were assessed in two phases: the acute phase (0–10 min post‐injection), during which the number of instances of paw licking was recorded, and the inflammatory phase (10–30 min post‐injection), during which the cumulative duration of paw licking was measured to reflect the inflammatory response of the surrounding tissue. This method provided a quantitative assessment of pain‐related behavior induced by formalin injection.

### 2.7 Writhing Test

The writhing test was conducted to assess nociceptive responses [30]. This test involved the intraperitoneal injection of irritant substances to induce visceral pain, characterized by acute, tonic, and diffuse nociceptive sensations mediated by spinal and supraspinal pathways. In this study, writhes were induced by administering 0.1 mL/10 g body weight of 0.6% acetic acid intraperitoneally. Five minutes post‐injection, the nociceptive response was evaluated by recording the number of abdominal writhes displayed by the animals over a 5‐min observation period. This method provided quantitative data on pain‐related behavior elicited by chemical stimulation of the peritoneal cavity.

### 2.8 Von Frey Filament Test

The paw withdrawal threshold (PWT) was assessed using the von Frey filament (VFF) test to evaluate mechanical nociception. Mice were individually placed on a mesh floor in transparent plastic boxes and allowed to acclimate. A series of VFFs with ascending forces (ranging from 0.02 to 1.4 g) were applied to the plantar surface of the hindpaw. Each filament was applied for 5–8 s, and the test was conducted five times with a 5‐min interval between stimulations. The PWT was determined as the lowest force that elicited a withdrawal response in at least three out of five stimulations. Withdrawal reflexes were identified by observable behaviors, including ipsilateral rear leg vibration, paw withdrawal, nibbling, or vocalization. This method provided a quantitative measure of mechanical pain sensitivity.

### 2.9 Hargreaves Test

The Hargreaves test was conducted to assess thermal nociception. Mice were placed in individual plastic boxes on a glass platform maintained at 30°C (IITC model 400, Woodland Hills, USA) to acclimate. A radiant heat source (Ugo Basile, Italy) with an intensity of 50 W was directed at the plantar surface of the hindpaw through the glass bottom. The paw withdrawal latency (PWL) was automatically recorded as the time from heat application to paw withdrawal. To prevent tissue damage, the maximum cutoff latency was set at 25 s. Each paw was tested at 15‐min intervals, and the average PWL was calculated from 3 to 6 trials per paw. This procedure provided a precise measure of thermal nociceptive sensitivity.

### 2.10 Immunofluorescence Staining

Immunofluorescence staining was performed to assess the localization and expression of specific proteins in tissue samples. Tissue sections were obtained from spinal cord or other relevant tissues, depending on the experimental model, and were fixed in 4% paraformaldehyde (PFA) for 15 min at room temperature. Following fixation, sections were washed with PBS and permeabilized using 0.3% Triton X‐100 in PBS for 10 min to allow for antibody penetration. Non‐specific binding sites were blocked by incubating the sections in 5% bovine serum albumin (BSA) for 1 h at room temperature. Primary antibodies targeting specific proteins were diluted in blocking buffer and applied to the sections overnight at 4°C. After primary antibody incubation, sections were washed three times in PBS, followed by incubation with appropriate secondary antibodies for 1 h at room temperature in the dark. Nuclei were counterstained with 4',6‐diamidino‐2‐phenylindole (DAPI) to visualize cell nuclei. Then sections were washed again with PBS and mounted on glass slides using a fluorescence mounting medium. The fluorescence signals were visualized and captured using a confocal microscope (LSM710, Zeiss, Oberkochen, Baden‐Würburg, Germany).

### 2.11 Real‐Time Polymerase Chain Reaction

Total RNA was extracted from the relevant tissue (spinal cord or cultured cells) using the RNeasy Plus Mini Kit (Qiagen, Germany) according to the manufacturer's instructions. Spinal cord tissues from the lesion epicenter (approximately 0.3 cm) were collected following euthanasia of the mice. The tissues were homogenized for RNA isolation. Total RNA was extracted using Trizol reagent (Takara, Shanghai, China) according to the manufacturer's protocol. Reverse transcription of RNA into complementary DNA (cDNA) was performed using a reverse transcription kit (R122‐01, Vazyme, Nanjing, China). Real‐time polymerase chain reaction (RT‐qPCR) was carried out using AceQ qPCR SYBR Green Master Mix (Q111‐02, Vazyme) on an RT‐PCR machine (Applied Biosystems, Carlsbad, CA, USA). The cycling conditions were as follows: an initial denaturation at 95°C for 30 s, followed by 40 cycles of 95°C for 10 s and 60°C for 30 s, with a final step at 65°C for 5 s. Primer sequences used in the experiments are listed in Table S1. All reactions were performed in triplicate to ensure accuracy and reproducibility.

### 2.12 Enzyme‐Linked Immunosorbent Assay

In this study, the enzyme‐linked immunosorbent assay (ELISA) was employed to quantify the concentration of specific biomarkers in samples. Briefly, 96‐well plates were coated with 100 μL of capture antibody diluted in buffer and incubated overnight at 4°C. After blocking with 1% BSA in PBS for 1 h at room temperature, 100 μL of sample or standard (prepared according to the manufacturer's instructions) was added to each well. Plates were incubated for 2 h at room temperature, followed by washing with PBS containing 0.05% Tween 20. A biotinylated detection antibody was then added to each well and incubated for 1 h. After washing, 100 μL of horseradish peroxidase‐conjugated streptavidin was added and incubated for 30 min. Absorbance was measured using a microplate reader. All assays were performed in duplicate, and results were compared with a standard curve.

### 2.13 LDH Detection

Lactate dehydrogenase (LDH) release was assessed to measure cytotoxicity in BV2 cells. BV2 cells were seeded in 96‐well plates at a density of approximately 1 × 10^4^ cells per well. LDH activity in the cell culture supernatants was quantified using the LDH assay kit (Servicebio, G1610‐100 T). The working solution was prepared by mixing the substrate mixture, iodine‐nitrotetrazolium substrate solution, dilution buffer, and enzyme solution in the volume ratios specified by the manufacturer's protocol. The working solution was then added to the wells containing the supernatants. Following incubation at 37°C for 30 min, the optical density (OD) of each well was measured at 490 nm using a microplate reader. The results provided a quantitative measure of LDH release, indicating the extent of cell membrane damage.

### 2.14 PI Staining

BV2 cells were seeded in 24‐well plates at a density of approximately 2 × 10^5^ cells per well and allowed to adhere. Cells were then stained with propidium iodide (PI, red) and DAPI to assess cell viability and nuclear morphology. Staining was performed by incubating the cells with the dyes for 30 min at room temperature, protected from light. After staining, the cells were visualized and imaged using a confocal microscope to evaluate the distribution and intensity of PI and DAPI signals. This method provided qualitative and quantitative insights into cell death and nuclear integrity.

### 2.15 Differentially Expressed Genes Analysis

The publicly available dataset GSE93561 was utilized to identify differentially expressed genes (DEGs) between normal (sham group) and injured (SCI group) spinal cord tissues. Gene expression profiles were retrieved and analyzed. DEGs were identified by comparing gene expression levels between the sham and SCI groups using a threshold of |log2 fold change| > 1 and an adjusted *p*‐value < 0.05. A heatmap was generated to visualize the clustering patterns of upregulated and downregulated genes across the groups. To further highlight the gene expression changes, a volcano plot was constructed, pinpointing genes with significant differential expression.

### 2.16 Statistical Analysis

Data analysis was performed by investigators blinded to the group assignments to ensure objectivity. Statistical analyses were carried out using GraphPad Prism (Version 10.0.3; GraphPad Software, Boston, MA, USA). To assess the distribution of continuous variables, the Shapiro–Wilk test was applied. If the data followed a normal distribution, comparisons between two groups were performed using Student's t‐test, and comparisons among multiple groups were conducted using one‐way analysis of variance (ANOVA) followed by Tukey's post hoc test. For non‐normally distributed data, the Mann–Whitney *U* test was used for two‐group comparisons, and the Kruskal‐Wallis test followed by Dunn's post hoc test was applied for multiple‐group comparisons. Statistical significance was set at *p* < 0.05. Data are presented as mean ± standard deviation (SD), and graphical representations were prepared to illustrate the analyzed outcomes.

## 3 Results

### 3.1 MS4A7 Is Significantly Upregulated in SCI and Inflammatory Stimuli

**FIGURE 1:**
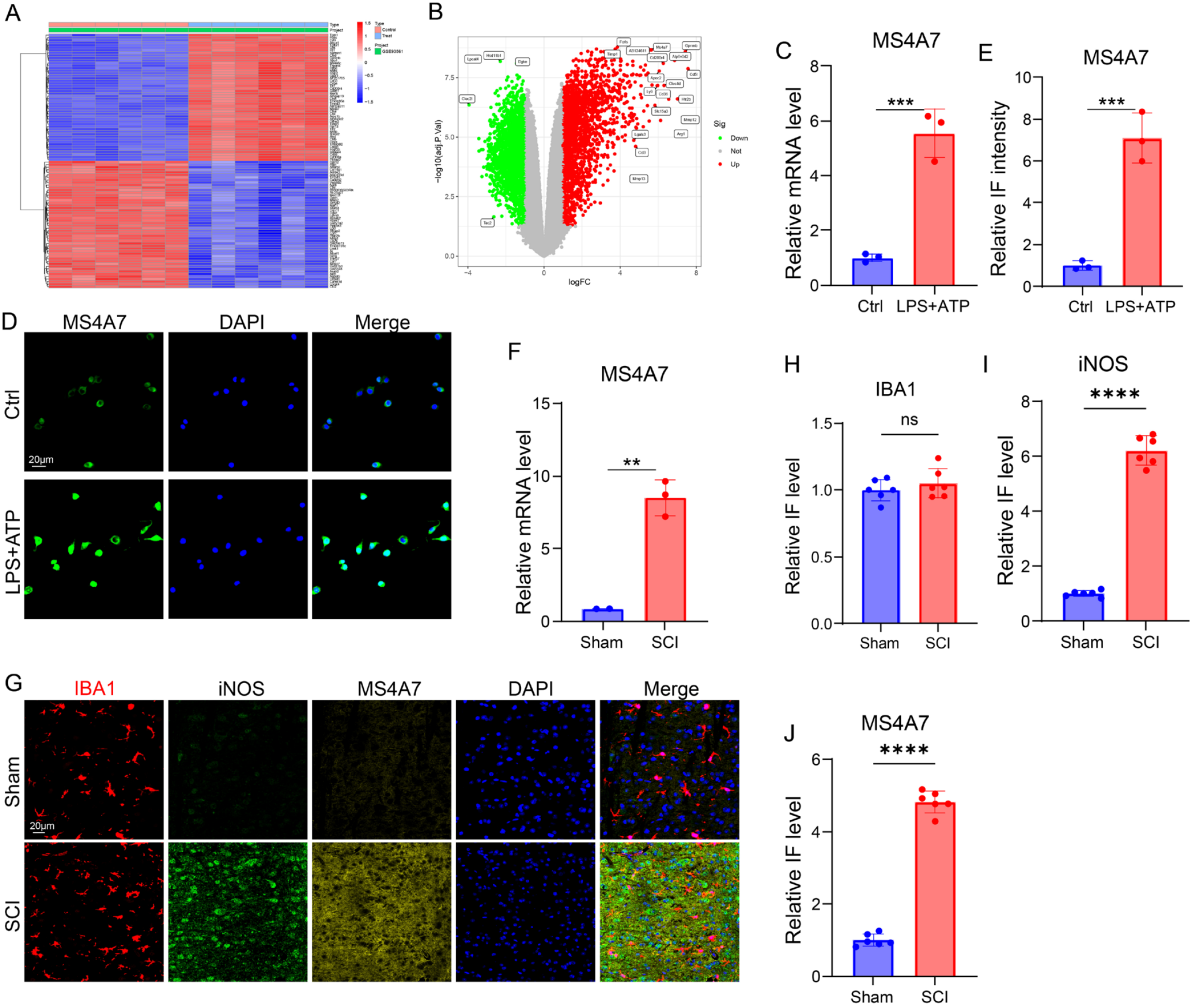
Transcriptomic and protein‐level analysis reveals MS4A7 upregulation in SCI and inflammatory stimuli. (A) Heatmap showing differentially expressed genes (DEGs) between sham and SCI groups based on dataset GSE93561. Distinct clusters of upregulated and downregulated genes are observed, with MS4A7 identified among the significantly upregulated genes in the SCI group. (B) Volcano plot of DEGs between sham and SCI groups. MS4A7 is prominently upregulated. (C) RT‐qPCR analysis of MS4A7 mRNA levels in BV2 cells treated with lipopolysaccharide (LPS) and ATP versus untreated controls (Ctrl), showing significant upregulation (*n* = 3 per group). (D, E) Immunofluorescence images showing MS4A7 protein expression and localization in BV2 cells. Weak MS4A7 expression is observed in untreated controls, while LPS + ATP treatment significantly enhances MS4A7 expression (*n* = 3 per group). (F) RT‐qPCR analysis of MS4A7 mRNA levels in SCI and sham spinal cord tissues, confirming transcriptional upregulation in the SCI group (*n* = 3 per group). (G) Immunostaining for IBA1 (a microglial marker), iNOS, and MS4A7 between sham and SCI groups (*n* = 6 per group). (H–J) Quantification of IBA1, iNOS, and MS4A7 immunofluorescence intensity (*n* = 6 per group). Statistical significance was determined: **p < 0.01, ***p < 0.001, ****p < 0.0001, ns = not significant.

SCI is characterized by neuroinflammation, and targeting excessive inflammatory responses is a useful method for treating SCI. Therefore, we tended to figure out the potential genes that play pivotal roles in modulating inflammation. The dataset GSE93561 was used to analyze the differentially expressed genes between normal and injured spinal cord tissues, in which a mice SCI model was established to investigate the mechanism of SCI progression [31]. The heatmap illustrates the differential expression of genes between the sham group and the SCI group, showing distinct clusters of upregulated and downregulated genes (Figure 1A). Among the differentially expressed genes, an inflammation modulation‐associated gene, MS4A7, is significantly upregulated in SCI conditions compared to sham controls. The volcano plot highlights the differential expression profile of genes, with MS4A7 prominently upregulated, confirming MS4A7 as a candidate gene of interest in the SCI group due to its substantial upregulation (Figure 1B). Semiquantitative mRNA levels of MS4A7 demonstrated a significant increase in MS4A7 mRNA levels in BV2 cells treated with lipopolysaccharide (LPS) and ATP compared to untreated controls (Ctrl), suggesting that MS4A7 is responsive to inflammatory stimuli (Figure 1C). MS4A7 protein levels and localization were visualized using immunofluorescence. Under control conditions, weak expression of MS4A7 was observed, while treatment with LPS + ATP significantly enhanced MS4A7 expression (Figure 1D,E). RT‐qPCR reveals significantly elevated MS4A7 mRNA levels in the SCI group compared to sham control, confirming transcriptional upregulation of MS4A7 in response to SCI (Figure 1F). Immunostaining for IBA1, a microglia marker, shows no significant difference in overall IBA1 fluorescence intensity between sham and SCI groups (Figure 1G,H). The level of iNOS was dramatically raised in the SCI group, indicating pro‐inflammatory activation, and the MS4A7 IF intensity was also remarkably upregulated after injury (Figure 1G,I,J). The results collectively demonstrate that MS4A7 is significantly upregulated in response to SCI and inflammatory stimuli (LPS + ATP), highlighting MS4A7 as a potential regulator of the inflammatory response in microglia and a candidate for further investigation in SCI‐related pathophysiology.

### 3.2 Knockdown of MS4A7 Enhances Locomotor Recovery and Alleviates Pain‐Related Behaviors Following SCI

**FIGURE 2:**
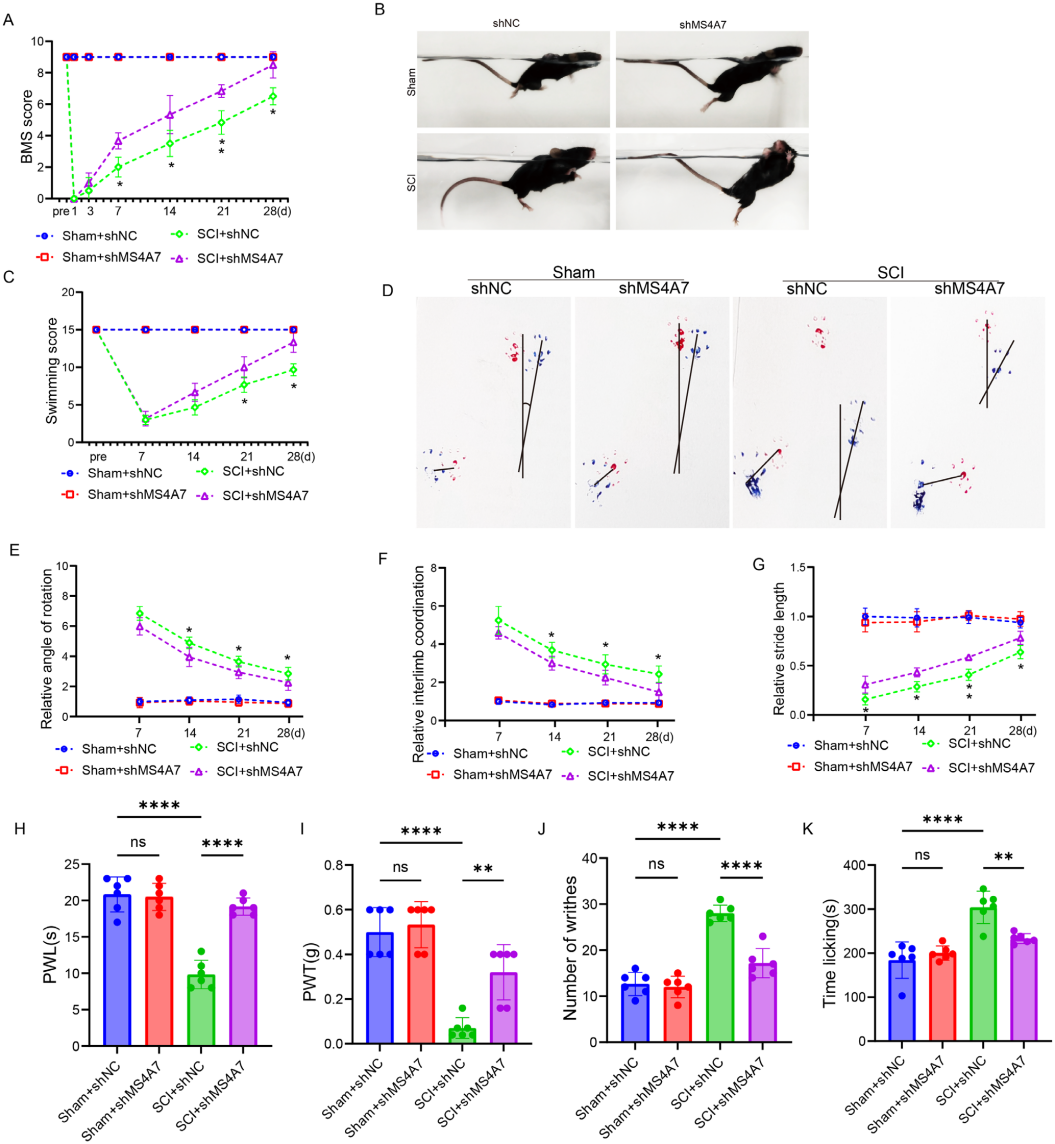
MS4A7 knockdown promotes motor functional recovery and reduces pain‐related behaviors in SCI mice. (A) BMS locomotor scores in sham and SCI mice with or without MS4A7 knockdown (shNC or shMS4A7). Both SCI + shNC and SCI + shMS4A7 groups exhibit significantly reduced scores at day 1 post‐SCI compared to sham controls. SCI + shMS4A7 mice show significantly improved recovery of BMS scores compared to SCI + shNC mice. (B, C) Swimming assessment reveals better limb movement and body coordination in SCI + shMS4A7 mice compared to SCI + shNC, with quantification of swimming scores. (D) Footprint analysis showing gait patterns, stride lengths, and hindlimb placement. MS4A7 knockdown improves hindlimb coordination and stride regularity post‐SCI. (E) Relative angle of rotation analysis demonstrates enhanced coordination and stability in SCI + shMS4A7 mice compared to SCI + shNC. (F) Hindlimb coordination scores reveal improved inter‐limb coordination in SCI + shMS4A7 mice compared to SCI + shNC. (G) Quantification of relative stride length, showing significantly longer strides in SCI + shMS4A7 mice compared to SCI + shNC. (H) Paw withdrawal latency (PWL) analysis indicates a significant restoration of thermal nociceptive thresholds in SCI + shMS4A7 mice compared to SCI + shNC. (I) Paw withdrawal threshold (PWT) analysis demonstrates significantly higher mechanical nociceptive thresholds in SCI + shMS4A7 mice, suggesting alleviation of mechanical hyperalgesia. (J) Writhing test quantifies visceral pain behaviors, showing significantly reduced writhes in SCI + shMS4A7 mice compared to SCI + shNC. (K) Formalin test reveals decreased time spent licking in SCI + shMS4A7 mice compared to SCI + shNC, indicating reduced pain‐related behaviors. Data represent mean ± SD (*n* = 6 per group). Statistical significance was determined: *p < 0.05, **p < 0.01, ****p < 0.0001, ns = not significant.

Spinal cord is the central nervous system, and the injury in T10 often results in impaired locomotor function and sensitized pain sensation. The results of BMS score showed that both SCI + shNC and SCI + shMS4A7 groups showed dramatically lowered BMS score compared to the sham‐operated groups (Sham + shNC and Sham + shMS4A7) at Day 1, indicating normal locomotion without SCI and successful SCI model establishment (Figure 2A). In addition, in the SCI + shMS4A7 group, the recovery of BMS scores was significantly restored compared to the SCI + shNC group, suggesting that MS4A7 knockdown promoted motor functional recovery after SCI (Figure 2A). Swimming ability is also an important way for assessing mice locomotor function. The SCI + shMS4A7 mice displayed improved limb movement and body coordination compared to the SCI + shNC group, demonstrating the critical role of MS4A7 in regulating post‐SCI motor function (Figure 2B). Swimming scores, an indicator of motor recovery, show that SCI + shMS4A7 mice had significantly poorer swimming performance compared to SCI + shNC mice (Figure 2B,C). Gait patterns visualized through foot‐printing reveal disrupted hindlimb placement and irregular stride patterns in SCI + shNC mice compared to the SCI + shMS4A7 group (Figure 2). The relative angle of rotation analysis indicates a significant increase in coordination and stability in SCI + shMS4A7 mice compared to the SCI + shNC group, emphasizing the impact of MS4A7 knockdown on rotational movements after SCI (Figure 2E). Relative hindlimb coordination analysis demonstrates that SCI + shMS4A7 mice exhibited substantial improvement in inter‐limb coordination compared to the SCI + shNC group (Figure 2F). The results of relative stride length show that SCI + shMS4A7 mice had a longer stride length compared to SCI + shNC mice (Figure 2G). What is more, pain‐related behaviors were evaluated. SCI significantly reduced PWL, and the SCI + shMS4A7 group showed significantly restored PWL compared to the SCI + shNC group, suggesting that MS4A7 knockdown improved thermal hyperalgesia (Figure 2H). The PWT analysis indicates a significant raise in mechanical nociceptive thresholds in SCI + shMS4A7 mice compared to SCI + shNC, suggesting an improvement of mechanical hyperalgesia upon MS4A7 knockdown (Figure 2I). In the writhing test, a marker of visceral pain, SCI + shMS4A7 mice exhibited a significantly lower number of writhes compared to SCI + shNC, demonstrating that MS4A7 knockdown alleviates pain perception after SCI (Figure 2J). The time spent licking in a formalin test was significantly decreased in the SCI + shMS4A7 group compared to SCI + shNC, indicating a decrease in pain‐related behaviors due to MS4A7 knockdown (Figure 2K). These findings collectively highlight the critical role of MS4A7 in modulating functional recovery, gait stability, and pain‐related behaviors following SCI. Knockdown of MS4A7 significantly improves locomotor recovery and alleviates mechanical and visceral pain, suggesting that MS4A7 may influence inflammatory or neural repair processes post‐SCI.

### 3.3 MS4A7 Silencing Promotes M2 Polarization of Microglia and Suppresses Pro‐Inflammatory Responses in SCI

**FIGURE 3:**
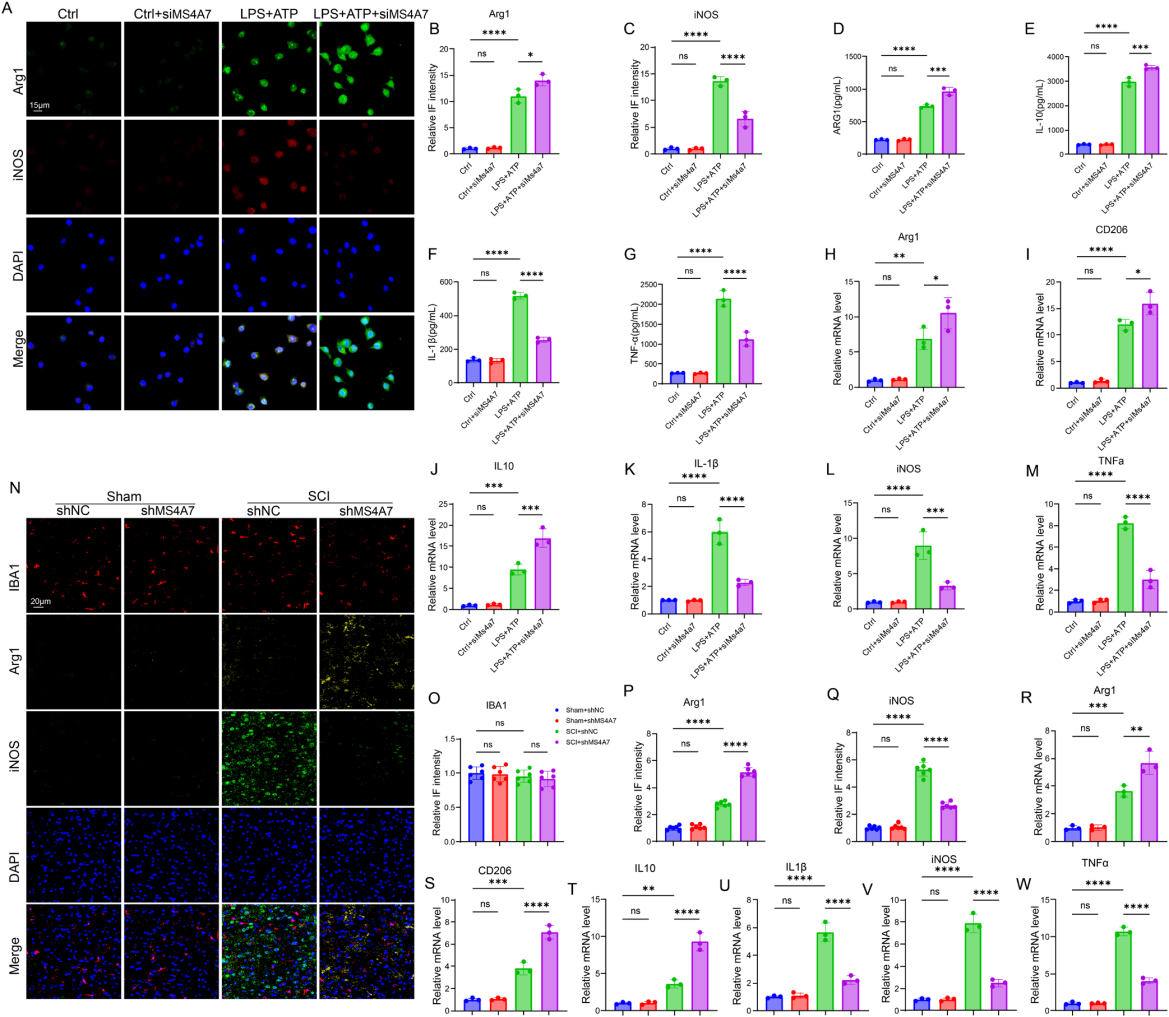
MS4A7 silencing enhances M2 microglial polarization and suppresses pro‐inflammatory cytokine production in vitro and in vivo. (A–C) Immunofluorescence staining of Arg1 (M2 marker) and iNOS (M1 marker) in BV2 microglial cells treated with LPS + ATP, with or without siMS4A7 (*n* = 3 per group). (D–G) ELISA results of polarization‐related cytokines (ARG1, IL‐10, IL‐1β, and TNF‐α) in BV2 cells (*n* = 3 per group). (H–M) mRNA expression analysis in BV2 cells reveals increased levels of Arg1, CD206, and IL‐10 and decreased levels of IL‐1β, iNOS, and TNF‐α in the LPS + ATP + siMS4A7 group, confirming M2 polarization and suppressed M1‐associated pro‐inflammatory markers (*n* = 3 per group). (N–Q) Immunostaining for IBA1, Arg1, and iNOS in SCI mice (*n* = 6 per group). (R–W) RT‐qPCR analysis of mRNA levels (Arg1, CD206, IL‐10, IL‐1β, iNOS, and TNF‐α) in SCI tissues (*n* = 3 per group). Data represent mean ± SD. Statistical significance was determined: *p < 0.05, **p < 0.01, ***p < 0.001, ****p < 0.0001, ns = not significant.

Microglia plays a pivotal role in modulating neuroinflammation in SCI. BV2 cells are a type of microglial cell derived from C57/BL6 murine [32]. To assess whether MS4A7 could affect microglia polarization, immunofluorescence staining of Arg1 (indicative of M2 polarization) and iNOS (indicative of M1 polarization) demonstrates the effects of MS4A7 silencing. The IF intensity of both Arg1 and iNOS was raised after the treatment of LPS + ATP (Figure 3A–C), indicating that inflammation was triggered and the anti‐inflammation mechanism was also activated. However, the downregulation of MS4A7 through siMS4A7 further elevated the level of Arg1 but lowered the level of iNOS (Figure 3A–C), showing that MS4A7 downregulation not only suppressed the M1 polarization but promoted the M2 polarization. Furthermore, the protein levels of M1 and M2 polarization indicators were detected through ELISA. The results showed that secreted levels of ARG1 and IL‐10 (anti‐inflammatory cytokines) were elevated in the LPS + ATP + siMS4A7 group, highlighting a shift toward an anti‐inflammatory, M2‐polarized state (Figure 3D,E). Inflammatory cytokines IL‐1β and TNF‐α were significantly reduced in the LPS + ATP + siMS4A7 group compared to the LPS + ATP group (Figure 3F,G), supporting the regulatory role of MS4A7 in suppressing inflammation. Additionally, the upregulation of mRNA levels of Arg1, CD206, and IL‐10, and the downregulation of mRNA levels of IL‐1β, iNOS, and TNF‐α confirm the promotion of M2 polarization (Figure 3H–M). To verify the role of MS4A7 in regulating microglia polarization in mice, the levels of polarization indicators such as Arg1 and iNOS were assessed. The fluorescence intensity of IBA1does not significantly differ between groups, indicating similar levels of microglial activation across conditions. However, the intensity of Arg1 is significantly increased in the SCI + shMS4A7 group compared to SCI + shNC, while iNOS intensity is reduced, consistent with enhanced M2 polarization (Figure 3N–Q). What is more, the mRNA levels of anti‐inflammatory cytokines Arg1, CD206, and IL‐10 are upregulated in SCI + shMS4A7 tissues, while pro‐inflammatory markers IL‐1β, iNOS, and TNF‐α are significantly reduced when compared to those in the SCI + shNC group (Figure 3R–W). These results collectively demonstrate that silencing MS4A7 promotes M2 microglial polarization, indicating that MS4A7 might play a critical regulatory role in modulating microglial polarization and inflammatory responses in SCI.

### 3.4 MS4A7 Overexpression Promotes M1 Polarization and Suppresses M2 Polarization in Neuroinflammatory Conditions

**FIGURE 4:**
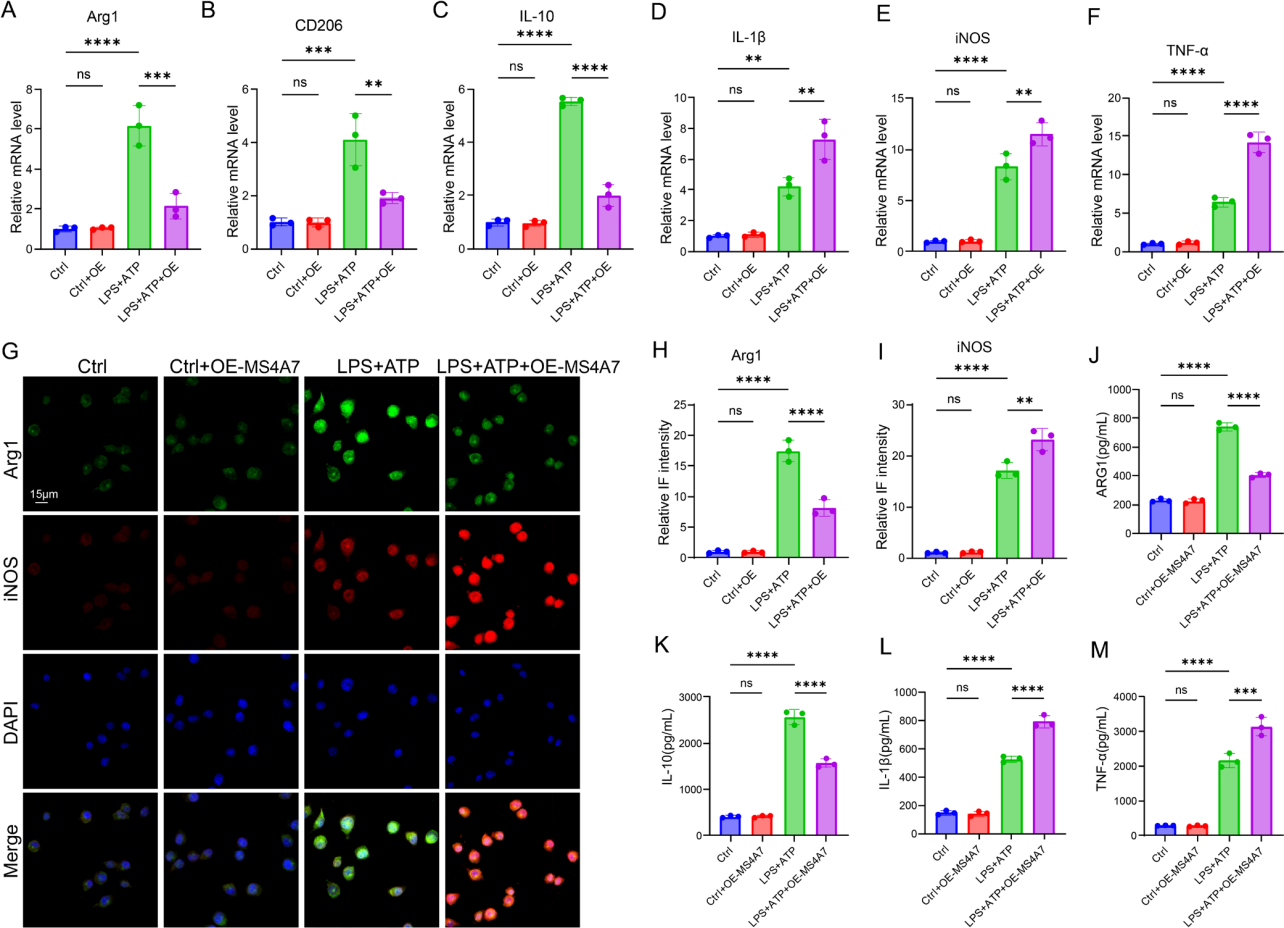
Overexpression of MS4A7 shifts microglial polarization toward the M1 phenotype and suppresses the M2 phenotype under neuroinflammatory conditions. (A–C) RT‐qPCR analysis of M2 polarization markers (Arg1, CD206, and IL‐10) in BV2 cells treated with LPS + ATP, with or without MS4A7 overexpression (OE‐MS4A7) (*n* = 3 per group). (D–F) RT‐qPCR analysis of M1 polarization markers (IL‐1β, iNOS, and TNF‐α) in BV2 cells (*n* = 3 per group). (G–I) Representative immunofluorescence images of Arg1 (M2 marker) and iNOS (M1 marker) in BV2 cells (*n* = 3 per group). (J, K) ELISA results showing significantly reduced secreted levels of ARG1 and IL‐10 (M2‐associated cytokines) in the OE‐MS4A7 group compared to LPS + ATP alone (*n* = 3 per group). (L, M) ELISA results of IL‐1β and TNF‐α (M1‐associated cytokines) demonstrate significantly elevated levels in the OE‐MS4A7 group (*n* = 3 per group). Data represent mean ± SD. Statistical significance was determined: **p < 0.01, ***p < 0.001, ****p < 0.0001, ns = not significant.

To further confirm the role of MS4A7 in promoting microglial M2 polarization in neuroinflammation conditions, we overexpressed MS4A7 in BV2 cells with or without LPS + ATP treatment. Overexpression of MS4A7 (OE‐MS4A7) significantly reduced the mRNA expression of M2 macrophage markers, including Arg1, CD206, and IL‐10, in BV2 cells stimulated with LPS and ATP (Figure 4A–C). This suggests a suppression of the M2 (anti‐inflammatory) phenotype by OE‐MS4A7. Conversely, OE‐MS4A7 significantly raised the mRNA levels of pro‐inflammatory cytokines, such as IL‐1β, iNOS, and TNF‐α, compared to LPS and ATP treatment alone, indicating a promotion of the M1 (pro‐inflammatory) phenotype (Figure 4D–F). Representative IF images illustrate decreased Arg1 expression and increased iNOS expression in BV2 cells treated with LPS + ATP + OE‐MS4A7 compared to LPS + ATP alone, further confirming the polarization toward the M1 phenotype (Figure 4G–I). Furthermore, the secreted levels of M1/2‐associated cytokines were detected. The M2‐associated cytokines ARG1 and IL‐10 were significantly reduced in the LPS + ATP + OE‐MS4A7 group compared to LPS + ATP alone, indicating suppressed anti‐inflammatory activity (Figure 4J,K). The levels of pro‐inflammatory cytokines IL‐1β and TNF‐α were significantly elevated in the OE‐MS4A7 group, demonstrating promotion of the inflammatory response (Figure 4L,M). Thus, the above results suggest a pivotal role for MS4A7 in regulating microglia polarization‐associated inflammation, potentially offering therapeutic insights for neuroinflammation in SCI.

### 3.5 MS4A7 Knockdown Mitigates NLRP3 Inflammasome Activation and Pyroptosis in Neuroinflammatory Conditions

**FIGURE 5:**
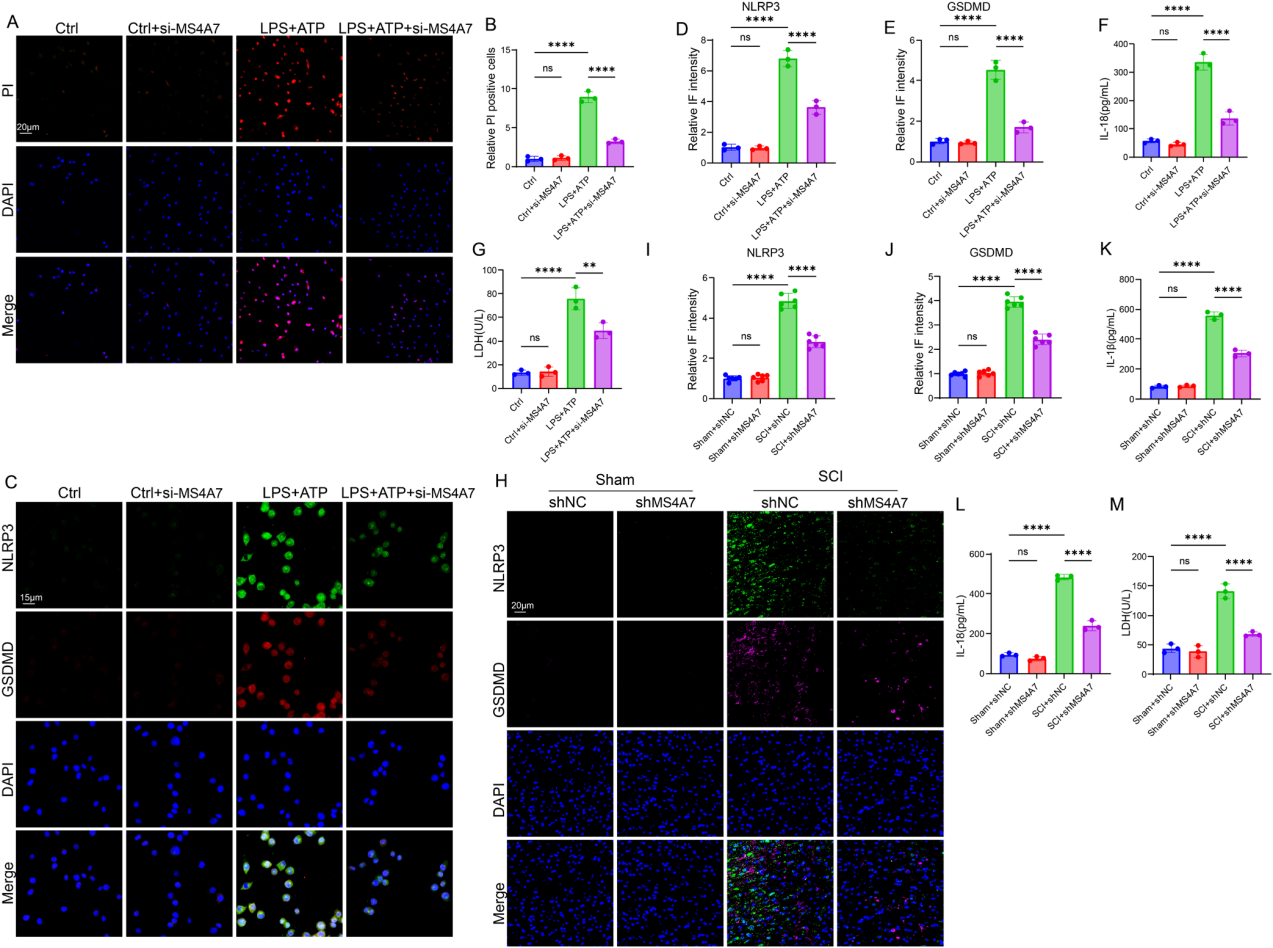
MS4A7 knockdown reduces NLRP3 inflammasome activation and pyroptosis in vitro and in vivo. (A, B) Propidium iodide (PI) staining assay in BV2 cells shows PI‐positive cells groups (*n* = 3). (C–E) Immunofluorescence staining of NLRP3 and gasdermin D (GSDMD) in BV2 cells (*n* = 3). (F) ELISA results for IL‐18 show significantly reduced cytokine secretion in the LPS + ATP + si‐MS4A7 group compared to LPS + ATP alone (*n* = 3). (G) LDH release assay in BV2 cells. (H–J) Immunofluorescence staining of NLRP3 and GSDMD in SCI tissues (*n* = 6). (K, L) ELISA results for IL‐1β and IL‐18 in SCI tissues show significant reductions in the SCI + shMS4A7 group compared to SCI + shNC (*n* = 3). (M) LDH release assessment (*n* = 3). Data represent mean ± SD. Statistical significance was determined: **p < 0.01, ****p < 0.0001, ns = not significant.

Recent studies revealed that M2‐like macrophage polarization was promoted by manipulating the proteasomal degradation of NLRP3 protein [33]. Importantly, Zhou et al. revealed that the inflammasome can be activated in an MS4A7‐dependent way [13]. Their study provided a novel insight into the mechanism of microglia polarization. Thus, we assessed whether MS4A7 could modulate the activation of NLRP3. The PI staining assay was conducted to assess cell membrane integrity as an indicator of pyroptotic cell death. The results demonstrate minimal PI‐positive cells in the Ctrl and Ctrl + si‐MS4A7 groups, while LPS + ATP significantly increased PI‐positive cells, indicating robust pyroptosis (Figure 5A,B). Knockdown of MS4A7 markedly reduced PI‐positive cells in the LPS + ATP group, suggesting that MS4A7 plays a critical role in mediating pyroptosis (Figure 5A,B). Immunofluorescence staining of NLRP3 and gasdermin D (GSDMD) was performed to examine inflammasome activation and pyroptotic signaling. The LPS + ATP condition showed pronounced NLRP3 and GSDMD activation compared to the control group (Figure 5C). However, knockdown of MS4A7 in the BV2 cells treated with LPS + ATP resulted in significantly reduced fluorescence intensities for both NLRP3 and GSDMD (Figure 5C–E). These findings indicate that MS4A7 is essential for inflammasome activation and subsequent pyroptotic signaling. The results of ELISA (IL‐1β and IL‐18) support this conclusion by showing a significant reduction in the IL‐18 level upon MS4A7 knockdown in the BV2 cells treated with LPS + ATP (Figures 3U and 5F), highlighting the downstream impact of MS4A7 on inflammasome‐mediated cytokine release. LDH release was measured as an additional marker of cell lysis and pyroptosis. The LPS + ATP group showed significantly elevated LDH levels compared to the control group, while MS4A7 knockdown reduced LDH release, reinforcing the role of MS4A7 in pyroptotic cell death (Figure 5G). The function of MS4A7 in modulating NLRP3 inflammasome and pyroptosis was further assessed through in vivo experiments. Immunofluorescence staining for NLRP3 and GSDMD reveals increased activation of these markers in the SCI + shNC group compared to the sham group, indicating that SCI induces robust pyroptosis (Figure 5H–J). Knockdown of MS4A7 (SCI + shMS4A7) significantly attenuated NLRP3 and GSDMD activation, suggesting that MS4A7 facilitates pyroptotic processes in the context of SCI (Figure 5H–J). The significant reduction in IL‐1β and IL‐18 secretion and LDH release in the SCI + shMS4A7 group further supports the role of MS4A7 in promoting inflammasome activation and pyroptosis during SCI (Figure 5K–M). The above findings collectively suggest that MS4A7 is a pivotal regulator of inflammasome activation and pyroptosis. Knockdown of MS4A7 effectively mitigates the activation of NLRP3, GSDMD, and associated pyroptotic markers in both in vitro and in vivo models.

### 3.6 MS4A7 Overexpression Amplifies NLRP3 Inflammasome Activation and Neuroinflammation

**FIGURE 6:**
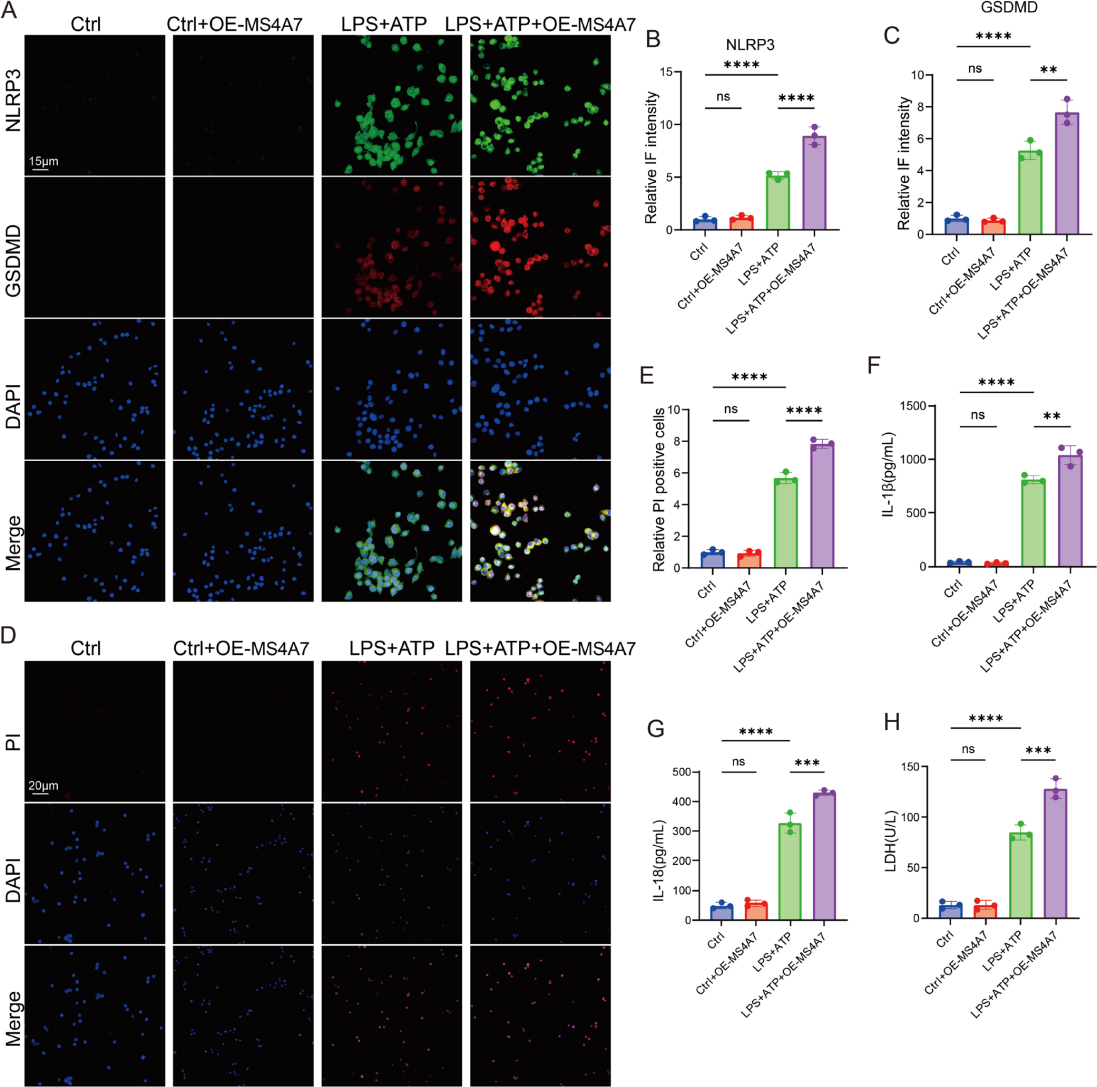
MS4A7 overexpression promotes NLRP3 inflammasome activation and exacerbates inflammation. (A–C) Immunofluorescence staining of NLRP3 and gasdermin D (GSDMD) in BV2 cells. (D, E) Propidium iodide (PI) staining assay. (F, G) ELISA results for IL‐1β and IL‐18. (H) LDH release assay shows that MS4A7 overexpression (LPS + ATP + OE‐MS4A7) further elevates LDH release compared to LPS + ATP alone. Data represent mean ± SD (*n* = 3 for in vitro experiments). Statistical significance was determined using one‐way ANOVA or Student's *t*‐test: **p < 0.01, ***p < 0.001, ****p < 0.0001, ns = not significant.

Immunofluorescence staining showed that no significant difference was observed in the NLRP3 expression between the ctrl group and the ctrl + OE‐MS4A7 group. However, compared to the ctrl group, LPS + ATP treatment significantly increases NLRP3 levels; overexpression of MS4A7 (LPS + ATP + OE‐MS4A7) further amplifies NLRP3 expression compared to LPS + ATP alone (Figure 6A,B). Immunofluorescence of GSDMD also showed that GSDMD expression is negligible in the ctrl and ctrl + OE‐MS4A7 groups; and LPS + ATP treatment markedly increases GSDMD level, which was further elevated by MS4A7 overexpression (LPS + ATP + OE‐MS4A7) (Figure 6A,C). Propidium iodide (PI) staining (red) is used to detect membrane‐permeable cells, indicative of cell death, likely through pyroptosis. LPS + ATP treatment increases PI‐positive cells, confirming pyroptosis induction. LPS + ATP + OE‐MS4A7 further elevates PI‐positive cells compared to LPS + ATP alone, suggesting that MS4A7 overexpression exacerbates pyroptotic cell death (Figure 6D,E). The release of IL‐1β and IL‐18, pro‐inflammatory cytokines, is also assessed. LPS + ATP induces robust IL‐1β and IL‐18 secretion, with significantly higher levels in the LPS + ATP + OE‐MS4A7 group compared to LPS + ATP alone. Similarly, LPS + ATP + OE‐MS4A7 further increases LDH level when compared with the LPS + ATP group, reinforcing the role of MS4A7 in promoting pyroptosis and inflammation. The above findings demonstrate that overexpression of MS4A7 significantly enhances NLRP3 inflammasome activation and the release of inflammatory mediators (IL‐1β and IL‐8) in an LPS + ATP‐induced pyroptotic model.

### 3.7 MS4A7 Promotes NLRP3 Inflammasome Activation via cGAS‐STING Pathway in Neuroinflammation

**FIGURE 7:**
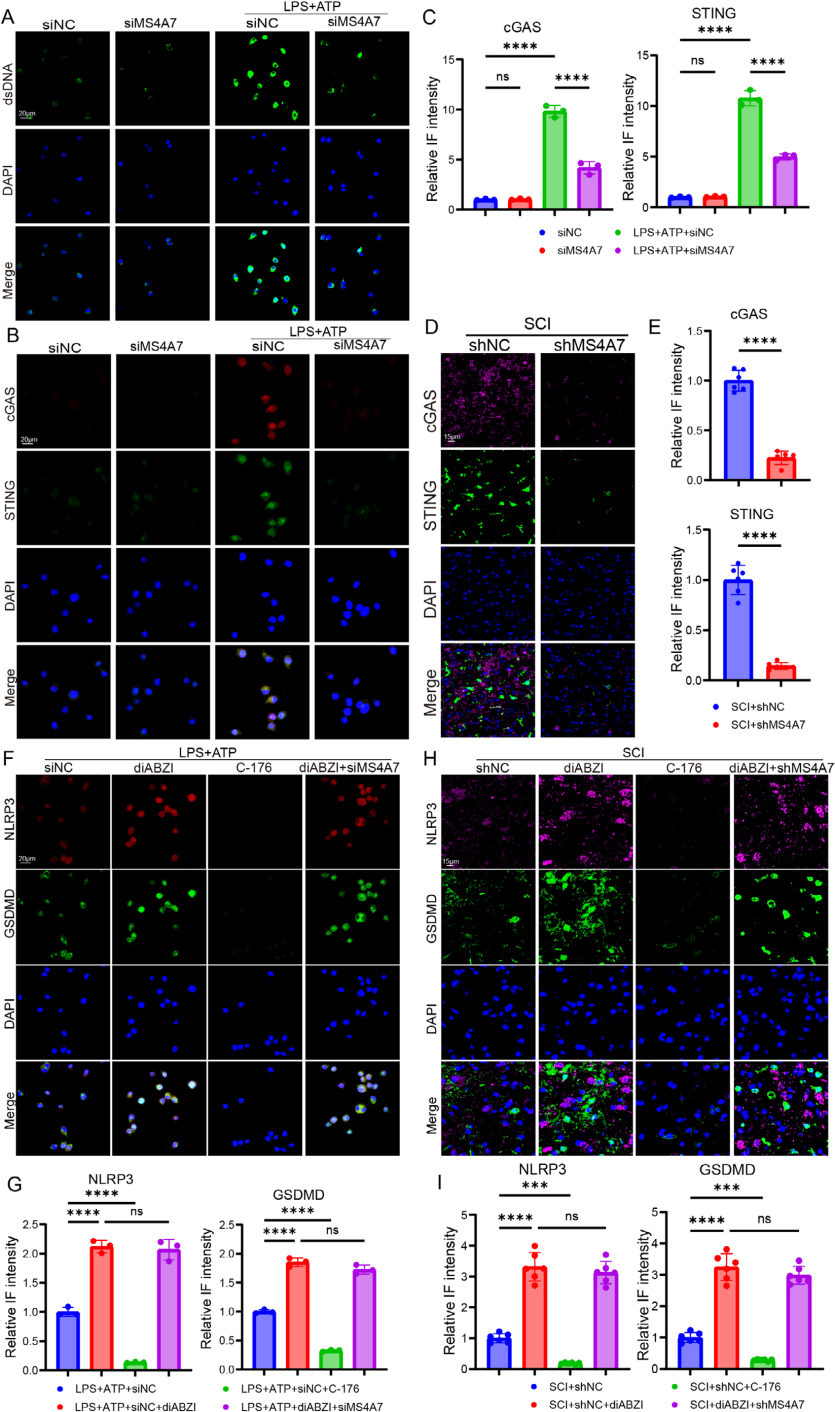
cGAS‐STING pathway mediates MS4A7‐induced NLRP3 inflammasome activation in vitro and in vivo. (A) Immunofluorescence for cytosolic dsDNA in BV2 cells (*n* = 3 per group). (B, C) Immunofluorescence staining and quantification of cGAS and STING in BV2 cells (*n* = 3 per group). (D, E) Immunofluorescence staining of cGAS and STING in SCI tissues (*n* = 6 per group). (F, G) Immunofluorescence staining for NLRP3 and GSDMD in BV2 cells (*n* = 3 per group). (H, I) Immunofluorescence staining for NLRP3 and GSDMD in SCI tissues (*n* = 6 per group). Data represent mean ± SD. Statistical significance was determined: ***p < 0.001, ****p < 0.0001, ns = not significant.

It has been reported that NLRP3 inflammasome activation was modulated by the cGAS‐STING pathway in some diseases including intervertebral disc degeneration and acute liver injury [34, 35, 36]. Thus, we speculated that cGAS‐STING pathway might play an important role in MS4A7‐mediated NLRP3 activation. We detected the level of cytosolic dsDNA and the activation of cGAS‐STING pathway after downregulating MS4A7. Immunofluorescence for dsDNA shows significantly lowered cytosolic DNA accumulation in the LPS + ATP + siMS4A7 group when compared to the LPS + ATP + siNC group (Figure 7A). Using JC‐1 staining, we observed that MS4A7 knockdown reduced mitochondrial membrane potential (ΔΨm) under LPS and ATP stimulation, as indicated by a decrease in red fluorescence aggregates and an increase in green fluorescence monomers (Figure S1A,B). To assess the impact of MS4A7 on redox balance, we measured glutathione (GSH) levels, ATP/ADP ratio, and cellular energy metabolism indicators. Compared to the LPS + APT+siNC group, LPS + APT+siMS4A7‐treated cells exhibited a significant increase in GSH level, implying decreased oxidative stress (Figure S1C). Moreover, siMS4A7 cells with LPS + APT treatment demonstrated a higher ATP/ADP ratio and ATP concentration, suggesting restored mitochondrial bioenergetics (Figure S1D,E). Consistently, the NAD+/NADH ratio was raised in siMS4A7‐treated cells, further confirming restored metabolic function (Figure S1F). These findings collectively suggest that MS4A7 deficiency plays a crucial role in maintaining mitochondrial integrity and cellular energy metabolism, particularly under inflammatory conditions. The suppression of dsDNA in the LPS + ATP + siMS4A7 group suggests that MS4A7 is involved in promoting cytosolic DNA release during LPS + ATP‐induced stress or inflammation. Immunofluorescence for cGAS and STING shows pronounced expression in the LPS + ATP + siNC group with compared to the LPS + ATP + siMS4A7 group (Figure 7B,C). Immunofluorescence and quantitative IF intensity for cGAS and STING in SCI tissues demonstrate marked reductions in the SCI + shMS4A7 group compared to the SCI + shNC group (Figure 7D,E). This further supports MS4A7's involvement in cGAS‐STING pathway activation during SCI‐induced inflammation.

To further investigate whether NLRP3 inflammasome activation could be modulated by the cGAS‐STING pathway, diABZI (STING activator) and C‐176 (STING inhibitor) were used to treat BV2 cells and mice. Immunofluorescence for NLRP3 and GSDMD in LPS + ATP‐treated BV2 cells shows elevated NLRP3 and GSDMD in the LPS + ATP + diABZI group, with reduced activation in the LPS + ATP + C‐176 group (Figure 7F,G). The above findings demonstrated that under the condition of LPS + ATP treatment, STING activation enhanced NLRP3 inflammasome activation and inhibition of the STING pathway suppressed the inflammasome, indicating that the NLRP3 inflammasome could be regulated by the cGAS‐STING pathway. What is more, the downregulation of MS4A7 (in the LPS + ATP + diABZI+siMS4A7 group) did not significantly affect the STING activator diABZI‐induced NLRP3 activation (Figure 7F,G), indicating that STING plays an essential role in MS4A7‐activated NLRP3 inflammasome. What is more, the immunofluorescence and quantitative IF intensity for NLRP3 and GSDMD in SCI tissue reveal similar trends to the in vitro results. NLRP3 and GSDMD activation were not markedly influenced in the SCI + diABZI + shMS4A7 group when compared to that in the SCI + diABZI group, confirming that MS4A7 contributes to inflammasome activation and pyroptosis in vivo through the cGAS‐STING pathway (Figure 7H,I).

### 3.8 NLRP3 Inhibition Reverses MS4A7‐Induced Microglial M1 Polarization and Promotes M2 Polarization in SCI

**FIGURE 8:**
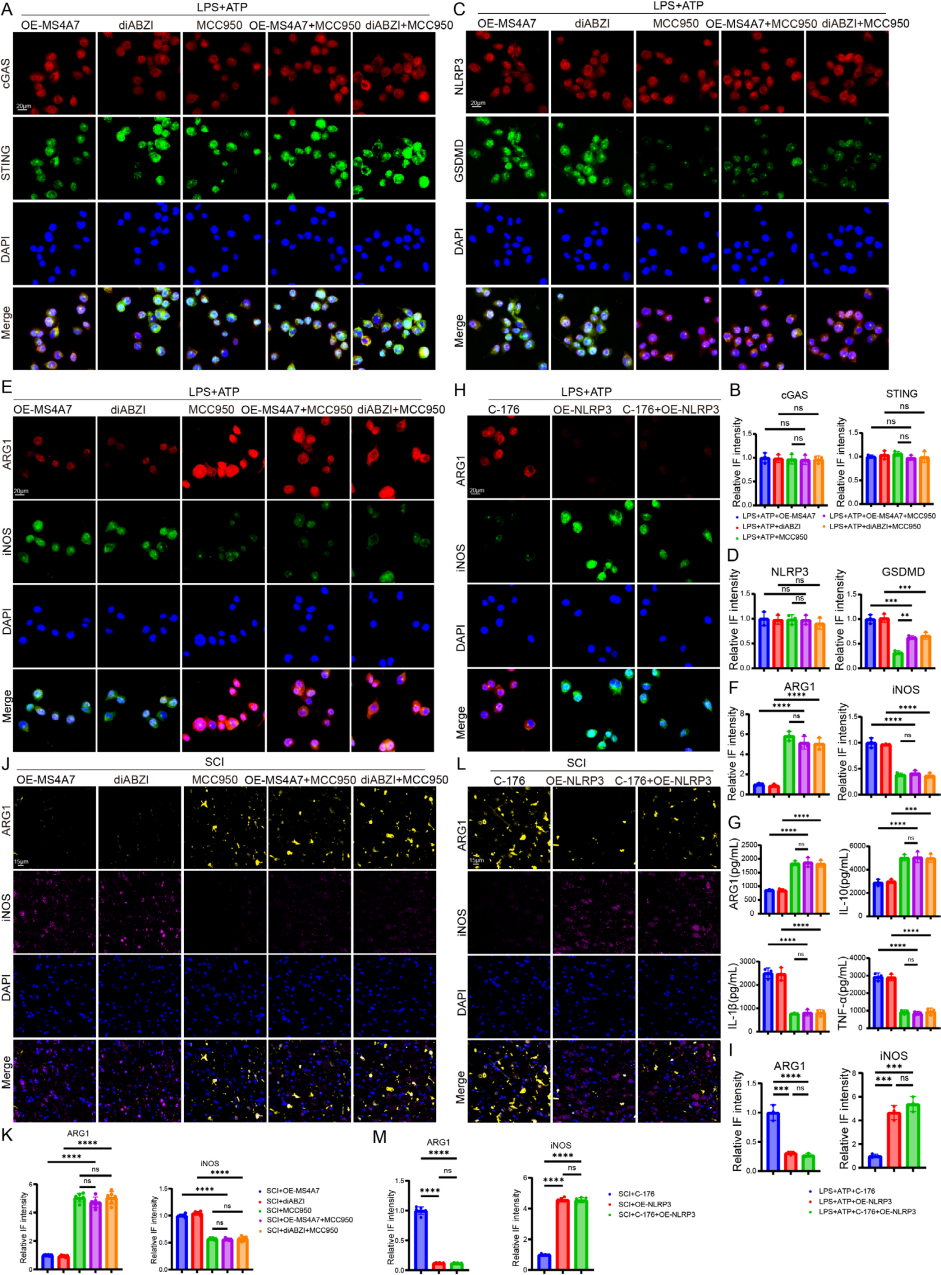
Pharmacological inhibition of NLRP3 inflammasome mitigates MS4A7‐induced pro‐inflammatory microglial polarization and promotes anti‐inflammatory responses. (A–D) Immunofluorescence staining and quantification of cGAS, STING, GSDMD, and NLRP3 in BV2 cells treated with LPS + ATP, diABZI, or OE‐MS4A7 with or without MCC950 (*n* = 3). (E, F) Immunofluorescence staining of ARG1 and iNOS in BV2 cells. (G) ELISA results for polarization‐related cytokines in BV2 cells (*n* = 3). (H, I) NLRP3 overexpression reduces the C‐176‐induced increase in ARG1 and reverses the decrease in iNOS in BV2 cells, further demonstrating the role of NLRP3 inflammasome activation in regulating polarization (*n* = 3). (J, K) Immunofluorescence staining of ARG1 and iNOS in SCI tissues (*n* = 6). (L, M) Immunofluorescence staining of ARG1 and iNOS in SCI mice treated with or without C‐176 or NLRP3 overexpression (*n* = 6). Data represent mean ± SD. Statistical significance was determined: **p < 0.01, ***p < 0.001, ****p < 0.0001, ns = not significant.

To further confirm whether NLRP3 inflammasome activation could promote microglia polarization in SCI, we treated BV2 cells and mice with MCC950 (a selective inhibitor of NLRP3, MCC950 intraperitoneal injection, 3 mg/kg/day) [37]. Immunofluorescence analysis demonstrates that compared to the LPS + ATP + OE‐MS4A7 group, the LPS + ATP + OE‐MS4A7 + MCC950 group showed no significant IF intensity values of cGAS, STING, and NLRP3 (Figure 8A–D). Similarly, IF intensity values of cGAS, STING, and NLRP3 in the LPS + ATP + diABZI +MCC950 group were also not significantly changed when compared to those in the LPS + ATP + diABZI group (Figure 8A–D). The above findings demonstrated that inhibiting NLRP3 activation via MCC950 could not influence the expression or activation of the cGAS‐STING pathway. However, as a downstream protein of NLRP3 inflammasome activation, the IF intensity of GSDMD was remarkably reduced by MCC950 with or without diABZI treatment or MS4A7 overexpression (Figure 8C,D), confirming that the NLRP3 inflammasome was suppressed by MCC950 and that the NLRP3 inflammasome was indeed activated by MS4A7 overexpression or cGAS‐STING activation. Furthermore, the indicators of polarization were detected. The IF intensity value of ARG1 in the LPS + ATP + OE‐MS4A7 + MCC950 group was remarkably elevated compared to that in the LPS + ATP + OE‐MS4A7 group; similarly, the IF intensity value of ARG1 in the LPS + ATP + diABZI + MCC950 group was remarkably elevated compared to that in the LPS + ATP + diABZI group (Figure 8E,F). However, the iNOS IF intensity was remarkably reduced in the cells treated with MCC950 (Figure 8E,F). The above results demonstrate that inhibiting NLRP3 could reverse microglial M1 polarization induced by MS4A7 overexpression or STING activation under the condition of inflammation. Then, the secretion levels of ARG1, IL‐10, TNF‐α, and IL‐1β were detected. The results showed that the levels of both ARG1 and IL‐10 were significantly raised by MCC950 when compared with other groups without MCC950 treatment (Figure 8G). The levels of TNF‐α and IL‐1β were remarkably lowered by MCC950 (Figure 8G). To further verify the role of NLRP3 in modulating microglial polarization, BV2 cells were treated with C‐176 (an inhibitor of STING) and/or NLRP3 overexpression. The results showed that NLRP3 overexpression reduced the C‐176‐induced increase in ARG1 and raised the decrease in iNOS (Figure 8H,I). In vivo studies were carried out to further verify the effect of NLRP3 inflammasome activation on microglial polarization. The IF intensity of ARG1 in both the SCI + OE‐MS4A7 + MCC950 group and the SCI + diABZI + MCC950 group was remarkably elevated compared to that in the SCI + OE‐MS4A7 group and the SCI + diABZI group; and the SCI + OE‐MS4A7 + MCC950 group and the SCI + diABZI+MCC950 group showed significantly lower iNOS IF intensity than the SCI + OE‐MS4A7 group and the SCI + diABZI group (Figure 8J,K), indicating that MCC950 treatment in SCI mice suppressed M1 polarization and promoted M2 polarization. However, no significant difference in ARG1 IF intensity was observed between the SCI + MCC950 group and the SCI + OE‐MS4A7 + MCC950 group, nor between the SCI + MCC950 group and the SCI + diABZI + MCC950 group (Figure 8J,K), confirming that NLRP3 inflammasome activation is downstream of MS4A7 and cGAS‐STING when modulating microglial polarization. In vivo experiments further confirmed the above findings, showing that NLRP3 overexpression reversed the C‐176‐induced M2 polarization (Figure 8L,M). These results demonstrate that MS4A7 exacerbates inflammation and promotes a pro‐inflammatory microglia phenotype via the cGAS‐STING‐NLRP3 axis. Pharmacological inhibition of NLRP3 effectively mitigates inflammation and favors a reparative, anti‐inflammatory phenotype by reducing iNOS expression and enhancing ARG1 expression. These findings provide a mechanistic basis for targeting the MS4A7 and cGAS‐STING‐NLRP3 axis in SCI to alleviate neuroinflammation and promote tissue repair.

## 4 Discussion

This study aimed to investigate the role of MS4A7 in modulating the inflammatory response and microglial polarization following SCI, focusing on its effects on the cGAS‐STING pathway and NLRP3 inflammasome activation. Our results demonstrated that MS4A7 knockdown significantly improved motor function and pain‐related behaviors post‐SCI through promoted anti‐inflammatory (M2) polarization of microglia and suppressed pro‐inflammatory (M1) polarization. Mechanistically, MS4A7 was shown to influence cGAS‐STING activation, which in turn regulates NLRP3 inflammasome‐mediated pyroptosis and inflammation. These findings shed light on the molecular mechanisms underlying SCI‐induced inflammation and suggest that targeting MS4A7 may represent a promising therapeutic strategy for SCI.

Microglial polarization is a critical determinant of neuroinflammation and recovery after SCI. This study found that MS4A7 expression was significantly upregulated following SCI and in inflammatory conditions induced by LPS and ATP treatment. Silencing MS4A7 enhanced M2 polarization, as evidenced by increased Arg1, CD206, and IL‐10 levels, while suppressing M1 polarization markers such as IL‐1β, iNOS, and TNF‐α. In contrast, MS4A7 overexpression promoted M1 polarization and inhibited M2 polarization, highlighting its pro‐inflammatory role. These findings are consistent with previous studies implicating MS4A7 in inflammatory regulation but extend the knowledge to demonstrate its specific involvement in SCI‐induced microglial polarization [9]. Recent research has increasingly recognized the pivotal role of microglial polarization in neuroinflammatory conditions, with studies reporting that shifting microglia toward an M2 phenotype significantly improves outcomes in neurodegenerative diseases, traumatic brain injury, and SCI [38]. For instance, recent investigations have shown that targeting molecular pathways such as PI3K‐Akt, STAT3, and NF‐κB can facilitate M2 polarization and reduce neuroinflammation [39]. Furthermore, advances in transcriptomics and proteomics have identified novel markers and regulators of microglial polarization, including specific miRNAs, long non‐coding RNAs, and epigenetic modifications, which provide insights into the intricate regulation of microglial states. The results of this study integrate with these findings by identifying MS4A7 as a key modulator of microglial polarization, particularly in the context of SCI. While prior research focused on general mechanisms underlying M1 and M2 polarization, this study highlights MS4A7's role in influencing specific cytokine profiles and phenotypic shifts in microglia. Importantly, the discovery of MS4A7's involvement adds a new dimension to the understanding of how the balance between pro‐inflammatory and anti‐inflammatory responses is regulated during SCI recovery. These results align with the emerging consensus that modulating polarization pathways—either through direct genetic manipulation or small‐molecule inhibitors—can significantly attenuate secondary injury [40, 41]. By linking MS4A7 to the broader context of microglial polarization research, this study underscores the potential for targeting MS4A7 as part of combination therapies aimed at mitigating neuroinflammation and promoting tissue repair.

The blood–brain barrier (BBB) plays a crucial role in maintaining central nervous system homeostasis, and its disruption following SCI exacerbates neuroinflammation by allowing peripheral immune cell infiltration and leakage of inflammatory mediators. Growing evidence suggests that pro‐inflammatory microglia can exacerbate BBB dysfunction by releasing cytokines such as TNF‐α and IL‐1β, which compromise endothelial barrier integrity [42]. As a membrane protein involved in immune regulation, MS4A7 may influence BBB permeability either directly through endothelial cell interactions or indirectly via microglia‐mediated inflammation. Although our present study did not directly assess BBB integrity, future research should focus on whether MS4A7 modulates vascular endothelial barrier function in SCI.

Inflammatory regulation plays a crucial role in the secondary injury phase of SCI, and multiple regulators have been implicated in modulating microglial activation and neuroinflammatory responses. Among these, well‐characterized molecules such as TREM2 and CD200R have been shown to modulate the balance between pro‐inflammatory and anti‐inflammatory microglial states, impacting neuronal survival and tissue repair. Similar to MS4A7, TREM2 has been identified as a critical regulator of microglial activation, influencing their ability to clear cellular debris and promote tissue homeostasis [43]. However, unlike MS4A7, which appears to drive a predominantly pro‐inflammatory response, TREM2 is generally associated with anti‐inflammatory and neuroprotective effects [44]. CD200R, another key regulator, acts as an inhibitory receptor suppressing excessive microglial activation, in contrast to MS4A7, which appears to enhance microglial pro‐inflammatory polarization [45]. Our findings suggest that MS4A7 may function similarly to classical pro‐inflammatory regulators such as NLRP3 and TNF‐α, both of which have been shown to exacerbate neuroinflammation in SCI models.

The cGAS‐STING pathway plays a pivotal role in sensing cytosolic DNA and mediating inflammatory responses. Recent research has emphasized the role of the cGAS‐STING pathway in various diseases, including cancer, autoimmune conditions, and neuroinflammatory disorders [46]. Recent studies have linked cGAS‐STING activation to several neuroinflammatory conditions, supporting its role as a critical regulator of inflammation in central nervous system disorders [47]. While most studies have focused on neurodegenerative diseases, a few emerging reports indicate that SCI‐induced tissue damage may trigger the release of cytosolic DNA, thereby activating the cGAS‐STING pathway [48, 49]. A recent study demonstrated that mitochondrial DNA leakage in nucleus pulposus cells activates cGAS‐STING‐NLRP3, exacerbating inflammation [36]. Furthermore, cGAS‐STING has been shown to enhance NLRP3 inflammasome activation in models of acute liver injury, indicating a potential mechanistic link in SCI [34]. In our study, we observed significant upregulation of cGAS and STING expression following SCI, with their inhibition leading to reduced neuroinflammation and improved functional recovery. These findings suggest that cGAS‐STING activation plays a crucial role in the secondary injury phase of SCI, making it a promising therapeutic target. What is more, studies have shown that activation of cGAS‐STING drives the production of type I interferons and pro‐inflammatory cytokines, contributing to the inflammatory milieu [50]. Study that revealed that DDIT3 blocked cGAS‐STING has highlighted the therapeutic potential of modulating this pathway in inflammatory conditions [51]. This present study revealed that MS4A7 knockdown reduced cGAS and STING expression, attenuating downstream inflammatory signaling, which is consistent with recent evidence showing that upstream regulators and scaffolding proteins can modulate cGAS‐STING activation [52]. In SCI models, the suppression of MS4A7 diminished cGAS‐STING activation, correlating with improved functional outcomes, including enhanced motor recovery and reduced pyroptotic cell death. Conversely, overexpression of MS4A7 amplified cGAS‐STING signaling, exacerbating inflammation and promoting secondary injury. Moreover, recent studies have identified connections between cGAS‐STING signaling and microglial activation, revealing its role in shifting microglia toward pro‐inflammatory phenotypes [22, 53]. The results of this study expand on these insights by demonstrating that MS4A7 serves as a novel modulator of cGAS‐STING activation in SCI, influencing microglial polarization and the inflammatory cascade. The findings highlight the interplay between MS4A7 and cGAS‐STING, adding complexity to the regulation of inflammation in SCI.

Activation of the NLRP3 inflammasome amplifies the inflammatory cascade in SCI, contributing to pyroptosis and secondary injury [2]. In the context of SCI, the activation of NLRP3 has been implicated in exacerbating tissue damage and inhibiting functional recovery [2, 54]. Recent advances have identified multiple upstream signals, such as mitochondrial dysfunction, reactive oxygen species (ROS), and cytosolic DNA, as key activators of NLRP3, with pathways like cGAS‐STING playing an increasingly recognized role in this regulation [36, 52]. This present study demonstrated that MS4A7 modulates NLRP3 inflammasome activation through the cGAS‐STING pathway. Knockdown of MS4A7 suppressed NLRP3 and GSDMD activation, reducing inflammatory cytokine release, consistent with recent findings that targeting upstream regulators of NLRP3 can effectively attenuate inflammation and pyroptosis in SCI. Conversely, MS4A7 overexpression enhanced inflammasome activation and downstream pyroptotic signaling, adding to the growing body of evidence that links excessive NLRP3 activation to worse outcomes in SCI. Interestingly, pharmacological inhibition of STING or NLRP3 effectively mitigated MS4A7‐induced inflammation, suggesting that the NLRP3 inflammasome acts downstream of MS4A7 and cGAS‐STING. This is supported by recent research demonstrating that cGAS‐STING can amplify NLRP3 activation in response to cytosolic DNA accumulation in conditions like intervertebral disc degeneration, acute liver injury, and insulin resistance [36, 52, 55]. These studies emphasize that cGAS‐STING serves as a critical upstream signal that primes or directly activates the NLRP3 inflammasome, particularly under inflammatory conditions. The findings of this study highlight a specific MS4A7‐dependent mechanism in SCI, where MS4A7 influences cGAS‐STING activation, which subsequently regulates NLRP3‐mediated microglial polarization. This provides a mechanistic understanding of how MS4A7 exacerbates neuroinflammation and secondary injury. By integrating the results of this study with current research advances, it becomes evident that targeting the MS4A7‐cGAS‐STING‐NLRP3 axis could be a promising therapeutic strategy for mitigating neuroinflammation, not only in SCI but also in a broader spectrum of inflammatory diseases.

Despite the encouraging results, the current study suffered from some limitations. First, while in vivo and in vitro models provided robust evidence, the translational potential of targeting MS4A7 in human SCI requires further validation. Second, the exact molecular interactions between MS4A7, cGAS‐STING, and NLRP3 remain partially elucidated, warranting deeper mechanistic studies. Third, the potential off‐target effects of pharmacological inhibitors used in this study need careful evaluation to confirm their specificity. Fourth, as a membrane‐bound protein, MS4A7 may be incorporated into EVs and exosomes, facilitating its transport between microglia and other central nervous system cells. Future investigations should explore whether MS4A7 is present in microglia‐derived exosomes and whether exosomal MS4A7 influences neuroinflammation and secondary injury responses in SCI.

## 5 Conclusions

In summary, this study identifies MS4A7 as a critical regulator of microglial polarization, cGAS‐STING activation, and NLRP3 inflammasome‐mediated microglia polarization in SCI. Silencing MS4A7 promotes an anti‐inflammatory environment, enhances functional recovery, and alleviates pain‐related behaviors in SCI models. These findings highlight the therapeutic potential of targeting MS4A7 and its downstream pathways to mitigate neuroinflammation and improve outcomes in SCI. Future research should focus on validating these mechanisms in clinical settings and exploring combinatory therapeutic approaches targeting MS4A7, cGAS‐STING, and NLRP3.

## Author Contributions

Xiangrui Li, Changsheng Li, and Junpeng Liu conceived the study, designed the experiments, and wrote the manuscript. Xiangrui Li performed the in vivo and in vitro experiments, including the SCI model, microglial polarization assessments, and data analysis. Youliang Deng contributed to the pathway analysis and data analysis. Junpeng Liu assisted with data collection and analysis. Fang Xing did the immunofluorescence assay. Xihua Lu and Zhen Zhang provided expert advice on the experimental design and participated in the interpretation of results. Changsheng Li supervised the study, designed the overall research strategy, and reviewed the manuscript. All authors read and approved the final manuscript.

## Conflicts of Interest

The authors declare no conflicts of interest.

## Supporting information


**Figure S1.** MS4A7 knockdown disrupts mitochondrial membrane potential and impairs cellular bioenergetics. (A) Representative fluorescence images of JC‐1 staining in siNC‐ and siMS4A7‐treated cells under basal conditions and following LPS + ATP stimulation. Scale bar, 15 μm. (B) Quantification of JC‐1 aggregate‐to‐monomer ratio, reflecting mitochondrial membrane potential changes. (C) Intracellular glutathione (GSH) levels were measured to assess oxidative stress. (D–E) ATP/ADP ratio and ATP concentration were evaluated to determine mitochondrial bioenergetic capacity. (F) NAD+/NADH ratio was analyzed to assess metabolic homeostasis. Data represent mean ± SD (*n* = 3 per group). Statistical significance was determined using one‐way ANOVA followed by Tukey’s post hoc test. *p < 0.05, **p < 0.01, ***p < 0.001, ****p < 0.0001, ns = not significant.

**Table S1.** Primers of the genes used in this study.

## Data Availability

The data that support the findings of this study are available on request from the corresponding author. The data are not publicly available due to privacy or ethical restrictions.
