# Supplementary material for: Role of MS4A7 in Regulating Microglial Polarization and Neuroinflammation in Spinal Cord Injury via the cGAS‐STING‐NLRP3 Axis
Source: CNS Neurosci Ther. 2025 Jun 16;31(6):e70390. doi: 10.1111/cns.70390 (PMC12168239; doi:10.1111/cns.70390)
Supplement: Supplementary file 1 — Figure S1. MS4A7 knockdown disrupts mitochondrial membrane potential and impairs cellular bioenergetics. (A) Representative fluorescence images of JC‐1 staining in siNC‐ and siMS4A7‐treated cells under basal conditions and following LPS + ATP stimulation. Scale bar, 15 μm. (B) Quantification of JC‐1 aggregate‐to‐monomer ratio, reflecting mitochondrial membrane potential changes. (C) Intracellular glutathione (GSH) levels were measured to assess oxidative stress. (D–E) ATP/ADP ratio and ATP concentration were evaluated to determine mitochondrial bioenergetic capacity. (F) NAD+/NADH ratio was analyzed to assess metabolic homeostasis. Data represent mean ± SD (n = 3 per group). Statistical significance was determined using one‐way ANOVA followed by Tukey’s post hoc test. *p < 0.05, **p < 0.01, ***p < 0.001, ****p < 0.0001, ns = not significant. [file CNS-31-e70390-s002.docx]

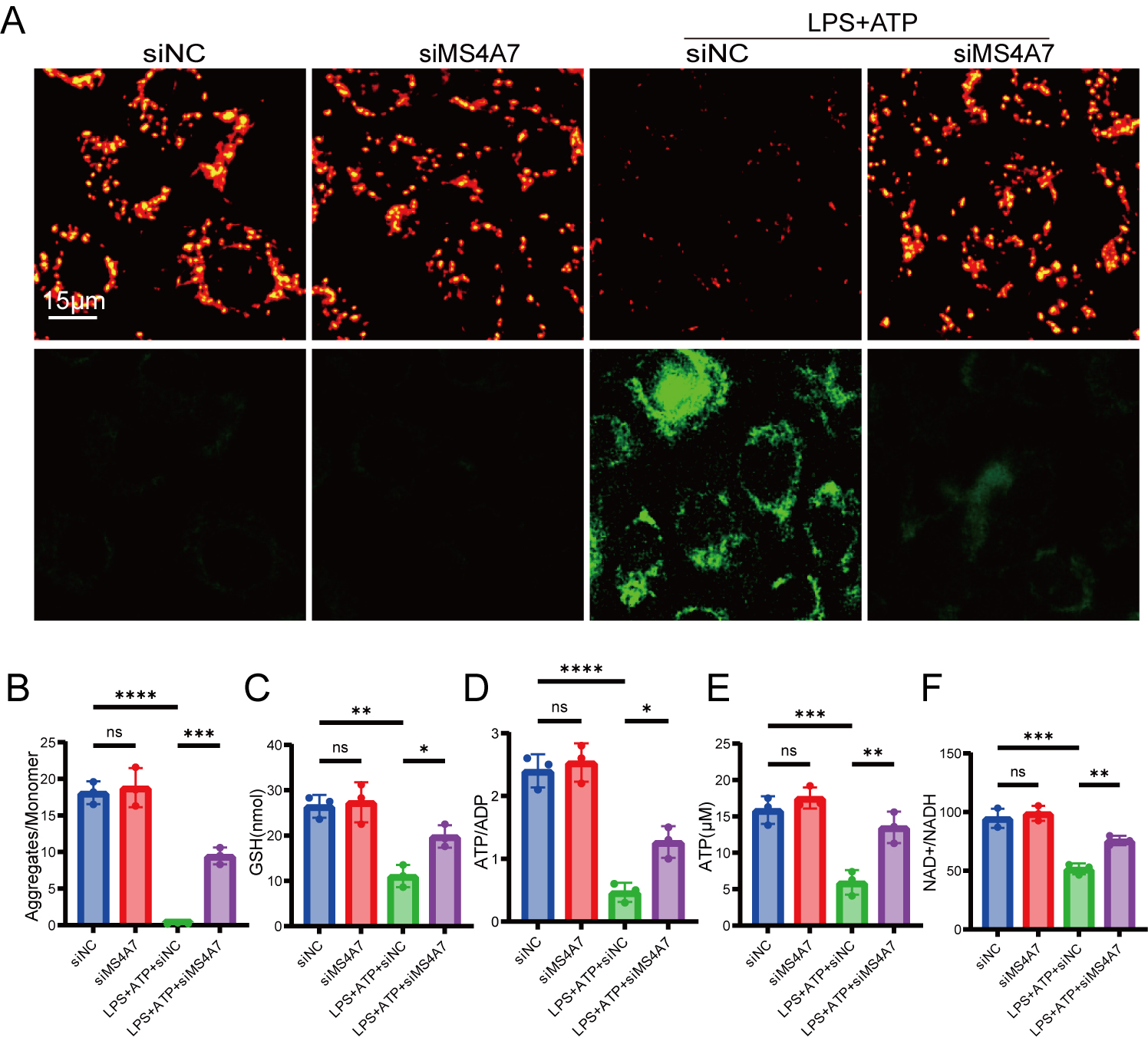


Figure S1. MS4A7 knockdown disrupts mitochondrial membrane potential and impairs cellular bioenergetics. (A) Representative fluorescence images of JC-1 staining in siNC- and siMS4A7-treated cells under basal conditions and following LPS+ATP stimulation. Scale bar, 15 μm. (B) Quantification of JC-1 aggregate-to-monomer ratio, reflecting mitochondrial membrane potential changes. (C) Intracellular glutathione (GSH) levels were measured to assess oxidative stress. (D–E) ATP/ADP ratio and ATP concentration were evaluated to determine mitochondrial bioenergetic capacity. (F) NAD+/NADH ratio was analyzed to assess metabolic homeostasis. Data represent mean ± SD (n = 3 per group). Statistical significance was determined using one-way ANOVA followed by Tukey’s post-hoc test. *p < 0.05, **p < 0.01, ***p < 0.001, ****p < 0.0001, ns = not significant.
