# Supplementary material for: Role of MS4A7 in Regulating Microglial Polarization and Neuroinflammation in Spinal Cord Injury via the cGAS‐STING‐NLRP3 Axis
Source: CNS Neurosci Ther. 2025 Jun 16;31(6):e70390. doi: 10.1111/cns.70390 (PMC12168239; doi:10.1111/cns.70390)
Supplement: Supplementary file 2 — Table S1. Primers of the genes used in this study. [file CNS-31-e70390-s001.docx]

Supplementary Table 1. Primers of the genes used in this study.

| Gene | Forward (5'–3') | Reverse (5'–3') |
| --- | --- | --- |
| MS4A7 | CTGTGAGGACAGCATCCCTG | ACTGTGGCTTCACTAGGCAC |
| Arg1 | TCATGGAAGTGAACCCAACTCTTC | TCAGTCCCTGGCTTATGGTTACC |
| iNOS | GTCCTACACCACACCAAACT | ATCTCTGCCTATCCGTCTC |
| CD206 | CAAGCGATGTGCCTACC | AATGCTGTGGATACTTGCC |
| IL-10 | GCTCTTACTGACTGGCATGAG | CGCAGCTCTAGGAGCATGTG |
| IL-1β | GAACAACAAAAATGCCTCGTGC | TGTCGTTGCTTGTCTCTCCTTGT |
| TNF-α | TACTGAACTTCGGGGTGATCG | TCCTCCACTTGGTGGTTTGC |
| GAPDH | TGTTTCCTCGTCCCGTAGA | ATCTCCACTTTGCCACTGC |
